# Engineered *Pseudomonas mirabilis*-Derived Outer Membrane Vesicles Targeting Bone Microenvironment to Improve Osteoporosis

**DOI:** 10.3390/biomedicines13040847

**Published:** 2025-04-02

**Authors:** Sanfu Lin, Chonggang Chen, Yuhui Zheng, Baofang Wu, Wenhua Wu

**Affiliations:** Department of Orthopedics, The Second Affiliated Hospital of Fujian Medical University, No. 34 Zhongshan Rd, Licheng District, Quanzhou 362000, China; 13600751728@139.com (S.L.); ccg05224@163.com (C.C.); 15060816999@139.com (Y.Z.); w15980272658@163.com (B.W.)

**Keywords:** osteoporosis, bacterial outer membrane vesicles, bone-targeting peptides, osteogenic differentiation, *Pseudomonas mirabilis*

## Abstract

**Introduction**: Osteoporosis (OP) is a prevalent condition marked by reduced bone density and a heightened risk of fractures. Current treatments often have side effects, underscoring the need for safer alternatives. Recent research highlights the significant role of gut microbiota and their metabolites in maintaining bone health. Notably, bacterial outer membrane vesicles (OMVs) have emerged as a promising platform due to their nanoscale sizes, low toxicity, drug-loading capabilities, and excellent biocompatibility. **Methods:** In this study, we developed a delivery system using OMVs derived from *Pseudomonas mirabilis* (PM). By anchoring bone-targeting peptides to the PM-OMVs membrane, we equipped these vesicles to deliver endogenous miRNAs to the bone microenvironment effectively. **Results and Discussion:** The bone-targeted PM-OMVs (PM-OMVs-BT) demonstrated exceptional bone-targeting abilities and exhibited a favorable safety profile in vivo. Additionally, LGG-OMVs-BT were successfully internalized by bone marrow stromal cells (BMSCs) without significant cytotoxicity, effectively promoting their osteogenic differentiation and mineralization. In conclusion, our study indicates that PM-OMVs-BT could offer a safe and effective treatment option for OP.

## 1. Introduction

Osteoporosis (OP) is a prevalent skeletal disorder characterized by reduced bone density, compromised bone architecture, and an increased risk of fractures and associated complications [1,2]. The pathophysiology of OP is primarily attributed to an imbalance between bone resorption and formation [3,4]. Additional factors such as race, gender, age, and diet also influence the bone mass and propensity to develop OP [5]. OP is prevalent among the elderly population and has emerged as a significant public health concern due to the global demographic aging [6]. Individuals diagnosed with OP are always confronted with elevated risk of fractures, which not only diminishes their quality of life but also imposes considerable healthcare burdens on society [7]. Currently, the most used therapeutics for clinical OP treatment include bisphosphonates, estrogen replacement therapy, denosumab, and calcium and vitamin D supplements [8,9]. However, these therapeutic drugs may lead to side effects such as atypical fractures, osteonecrosis of the jaw, cardiac thrombosis, hypocalcemia, or the development of certain cancers [10,11]. Therefore, novel therapeutics with targeting capacity to more effectively balance bone formation and resorption while reducing toxicity are in urgent need.

In recent years, the relationship between gut microbiota and bone health has garnered significant attention. A growing body of research indicates that gut bacteria participate in regulating bone metabolism through various pathways [12]. Gut bacteria affect bone metabolism indirectly or directly by influencing the immune system, hormone levels, and metabolic processes, and may play a significant role in the development and progression of OP [13]. Microbial products, such as short-chain fatty acids (SCFAs) and other bacterial metabolites, can regulate the function of bone metabolic cells, such as osteoclasts and osteoblasts, thereby maintaining bone mass balance [14]. Additionally, the regulation of the immune system by gut bacteria plays a key role in bone metabolism [15]. Dysbiosis of the gut microbiota can trigger inflammatory responses that disrupt the balance between bone formation and resorption, leading to OP [16].

OMVs have emerged as promising therapeutic agents in the treatment of OP. OMVs are membrane-bound particles secreted by bacteria, containing a variety of bioactive molecules such as proteins, lipids, and nucleic acids [17,18]. These vesicles can modulate immune responses, inflammation, and cellular functions, suggesting their potential role in bone metabolism regulation [19]. OMVs may serve as a strategy to circumvent the biocompatibility and large-scale production issues associated with synthetic nanomaterials. Many studies have demonstrated that OMVs can directly influence both osteoclasts and osteoblasts [20]. Chen et al. found that extracellular vesicles (EVs) derived from *Lactobacillus* spp. can enhance osteoblast differentiation and activity, potentially supporting bone formation [21]. Sapra et al. showed that EVs from *Bifidobacterium* spp. can modulate immune responses and reduce the production of pro-inflammatory cytokines, thereby alleviating the inflammation that typically exacerbates OP [22]. OMVs from PM have shown bone-protective effects by upregulating Abca1 expression and inducing mitochondrial-dependent apoptosis through the downregulation of miR-96-5p [23]. However, the lack of bone-targeting hinders the effective utilization of OMVs.

To address this limitation, recent studies have focused on engineering OMVs to enhance their therapeutic efficacy. Wang et al. modified PM-OMVs with bone-targeting peptides to enhance their therapeutic effects on OP [24]. Additionally, OMVs can serve as efficient drug carriers, improving the targeting and bioavailability of therapeutic agents in bone tissue, and offering a promising strategy for the diagnosis and treatment of OP. Liu et al. developed a drug delivery system using EVs secreted by *Lactobacillus rhamnosus* GG (LGG), isolated from the gut [25]. By anchoring bone-targeting peptides to the LGG-derived EVs, they improved the therapeutic efficacy for OP. Bone targeting LGG-EVs (BT-LGG-EVs) can deliver endogenous miRNAs to the bone microenvironment, promoting osteoblast differentiation and mineralization while inhibiting osteoclast formation, thus providing an innovative approach for managing OP. These studies highlight the potential of engineered OMVs as targeted therapies for OP.

In this study, bone-targeting peptides SDSSD were used to engineer OMVs, enhancing their therapeutic effects on OP. The PM-OMVs-BT effectively inhibited osteoclast differentiation and demonstrated strong targeting ability in mice, resulting in improved therapeutic outcomes for OP. These customized PM-OMVs-BT offer an advanced, safe, and effective treatment strategy for OP.

## 2. Materials and Methods

### 2.1. Strain Culture Medium and Cultivation

PM was purchased from ATCC 12453 and cultured in brain heart infusion broth. PM were cultured under aerobic conditions at 37 °C and 220 rpm. The fermentation broth of PM was used for subsequent isolation of PM-OMVs.

### 2.2. Isolation and Characterization of OMVs

Bacteria in the culture medium were first removed by low-speed centrifugation at 10,000× *g* for 1 h, and then the supernatant was filtered through a 0.22 μm sterile filter and concentrated using a 100 KDa ultrafiltration membrane. The filtrate was ultracentrifuged at 150,000× *g* for another 3 h to collect the OMVs, and the obtained OMVs were washed by PBS ultracentrifugation at 150,000× *g* for 3 h. All the OMVs separation processes were performed at 4 °C. The final resuspension was stored at −80 °C until use. The morphology of the isolated OMVs was characterized by transmission electron microscopy (TEM, Hitachi, Tokyo, Japan). The size and concentration of the isolated OMVs were characterized by nanoparticle tracking analysis (NTA, Malvern, NightSight, UK) in PBS buffer.

### 2.3. Synthesis of DSPE-PEG-Mal-Cys-SDSSD

A bone-targeting peptide SDSSD and 1,2-Distearoyl-sn-glycero-3-phosphoethanolamine (DEPE)-PEG-Maleimide (Mal) were synthesized by Wansheng Haotian Biological Technology (WSHT, Shanghai, China) and Xi’an Rixi Biological Technology Company (Ruixibio, Xi’an, China), respectively. They were dissolved in TCEP solution and HEPES buffer at 1 mg/mL. The peptide was then reacted with DSPE-PEG-Mal at a 1:1 molar ratio at room temperature for 24 h to form DSPE-PEG-Mal-Cys-SDSSD. This mixture was dialyzed against distilled water in a 3000 Da cutoff dialysis bag for 24 h, freeze-dried, and the resulting powder stored at −20 °C.

### 2.4. PM-OMVs-BT Construction

In order to prepare the PM-OMVs-BT, a gentle mixing was performed with 1 mL of PM-OMVs (10^14^ particles/mL), 9 mL of a 10 μM DSPE-PEG-Mal-Cys-SDSSD solution, and 10 mL of PBS. This mixture was then subjected to an overnight incubation at a temperature of 4 °C. Subsequently, the mixture underwent ultracentrifugation at a force of 150,000× *g* for a duration of 70 min, also maintained at 4 °C. Finally, the pellet was resuspended in PBS to ensure the complete removal of any residual DSPE-PEG-Mal-Cys-SDSSD.

### 2.5. Biophoton Imaging Analysis

All animal experiments were approved by the Animal Care and Use Committee of Fujian Medical University. For in vivo tracing experiments, 6-week-old C57BL/6 female mice (Slack Laboratory Animal Company, Shanghai, China) were randomly divided into two groups and treated with 100 μL of 1.0 × 10^9^ particles/mL Cy5-labeled PM-OMVs and PM-OMVs-PT through vein tail injection, respectively. The mice were killed 4 h, 8 h, and 16 h after intravenous injection, and their hearts, livers, spleens, lungs, kidneys, and bones were obtained and analyzed by fluorescence imaging using the Quickview 3000 system.

### 2.6. In Vivo Safety

The in vivo safety profile of PM-OMVs and PM-OMVs-PT is crucial for their potential future applications. To assess this, we conducted an in vivo biocompatibility study. Healthy mice were intravenously injected with 100 μL of 1.0 × 10^9^ particles/mL PM-OMVs, PM-OMVs-PT, or PBS (as the control) on a weekly basis for a total of eight weeks.

### 2.7. Cell Culture

Primary mouse BMSCs were isolated from long bones of the 6-week-old C57BL/6 female mice. The complete contents of the bone marrow cavity were rinsed and digested with enzyme buffer containing 4 g/L dispase, 3 g/L type I collagenase, and 1 U/mL DNAse I in HBSS buffer with calcium and magnesium for 15 min at 37 °C to obtain a single cell suspension. Subsequently, the enzymatic reaction was terminated with HBSS buffer with 2 mM EDTA and 2% fetal bovine serum (FBS). The cell suspension was cultured in a 5% CO_2_, 37 °C incubator for 72 h in α-MEM containing 20% FBS, 100 μg/mL streptomycin, and 100 U/mL penicillin. Non-adherent cells were gently removed, and adherent cells were cultured until the cell density reached about 90%. BMSCs from passages 3 to 5 were used for in vitro experiments.

### 2.8. Internalization Assay

To study the internalization of OMVs, 0.5 mg/mL of the fluorescent dye Cy5 was added to OMVs and incubated at room temperature for 20 min. The Cy5-labeled OMVs were washed with PBS and collected by ultracentrifugation at 100,000× *g* for 3 h. Subsequently, the Cy5-labeled OMVs were added to BMSCs and incubated for 6 h, and the cells were washed with PBS and fixed with 4% paraformaldehyde for 15 min. After washing with PBS again, the cells were stained with DAPI and incubated at 37 °C for 30 min. Confocal microscopy was used for observation and analysis.

### 2.9. Cell Viability

BMSCs were seeded in 96-well plates at a density of 2.0 × 10^3^/well and cultured overnight in α-MEM medium containing 10% FBS, 100 μg/mL streptomycin, and 100 U/mL penicillin. Cell viability was assessed using the CCK-8 cell proliferation assay. Specifically, 10 μL of CCK-8 solution was added to each well mixed thoroughly, taking care to avoid bubbles, and then incubated in a 37 °C incubator for 1 h. OD value was then measured at a wavelength of 450 nm on a microplate reader to assess cell viability. All samples were tested in duplicate 6 times, and the results are expressed as mean ± SEM.

### 2.10. Osteogenesis Induction and Evaluation

Bone marrow mesenchymal stem cells were cultured overnight in 24-well plates at a density of 2.0 × 10^4^ cells/well. The culture medium was α-MEM (10% fetal bovine serum, 100 μg/mL streptomycin, and 100 U/mL penicillin) and cultured overnight at 37 °C. Subsequently, the culture medium was replaced with an osteogenic differentiation medium (Oricell, Shanghai, China) to promote osteogenic differentiation of the cells. All culture media used were replaced every 2 to 3 days to keep the cell growth environment fresh and suitable. To evaluate the osteogenic effect of the cells, we used the alkaline phosphatase (ALP) activity assay. After 7 days of cell culture with 1.0 × 10^9^ particles/mL PM-OMVs and PM-OMVs-BT, the cells were stained with ALP using an ALP colorimetric kit (Beyotime, Nantong, China) according to the manufacturer’s guidelines to quantify ALP activity and evaluate the osteogenic ability of the cells.

### 2.11. Osteoclast Induction and Evaluation

Firstly, bone marrow cells were isolated from the long bones of newborn mice. Then, the bone marrow cells were co-cultured with M-CSF to promote the proliferation of osteoclast precursors. The M-CSF-dependent macrophages were transferred to a culture medium containing M-CSF, RANKL, as well as 1.0 × 10^9^ particles/mL PM-OMVs and PM-OMVs-BT for 7 days to induce osteoclast differentiation. Osteoclasts were identified using the TRAP (Tartrate-Resistant Acid Phosphatase) staining method, where mature osteoclasts appeared reddish-purple after staining. During the culture period, half of the culture medium was refreshed every 48 h to maintain a conducive environment for cell growth. Starting from the fourth day after the addition of RANKL, the cells were observed daily to ensure that experiments were conducted promptly after the osteoclasts had matured.

### 2.12. Animal Experiment

For in vivo experiment, 8-week-old C57BL/6 female mice (Slack Laboratory Animal Company, Shanghai, China) were randomly divided into four groups: Sham, OVX, OVX treated with PM-OMVs, and OVX treated with PM-OMVs-BT. Subsequently, injecting 1.0 × 10^9^ particles/mL of PM-OMVs or PM-OMVs-BT through the tail vein once a week for eight weeks. For in vivo tracking, mice were treated with 100 μL of 1.0 × 10^9^ particles/mL Cy5-labeled PM-OMVs and PM-OMVs-PT through vein tail injection, respectively. 

### 2.13. Micro-CT

One femur from each mouse was harvested and immediately fixed in a 4% paraformaldehyde solution. Serial sections were obtained from each femur, spanning from 0.15 mm below the growth plate to 0.4 mm towards the proximal direction. These sections were then subjected to Micro-CT scanning using a Skyscan 1276 device (Bruker Corporation, Billerica, MA, USA). The acquired scan data were subsequently processed to generate three-dimensional (3D) renderings of the metaphyseal and trabecular bone regions. These 3D reconstructions were meticulously analyzed using the Data Viewer 1.5.0.0 software to calculate a range of bone-related parameters.

### 2.14. Statistical Analysis

All data are presented as mean ± standard deviation, and statistical analysis was performed using one-way ANOVA and *t*-test between the two groups using GraphPad Prism 9. NS, not statistically significant, * *p* < 0.05, ** *p* < 0.01, *** *p* < 0.001.

## 3. Results

### 3.1. Design and Characterization of Engineered PM-OMVs-BT

**Figure 1:**
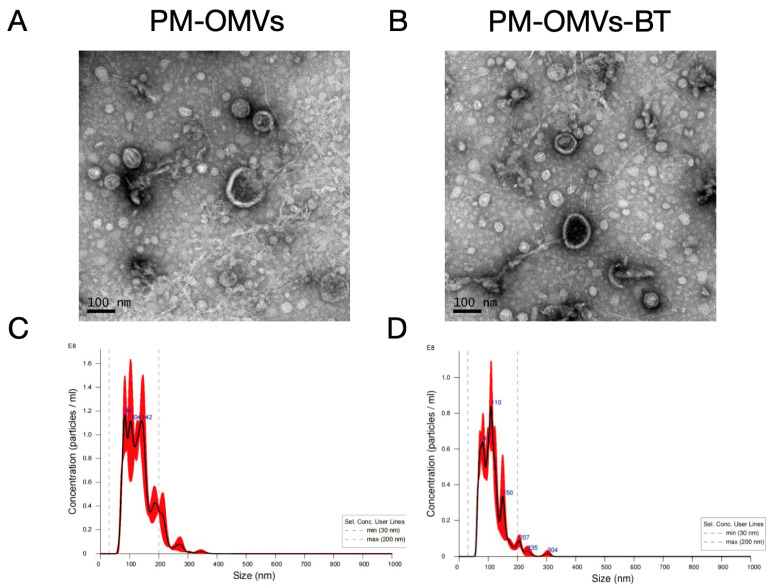
Characterization of engineered PM-OMVs-BT: (**A**) TEM image of PM-OMVs. Scale bar = 100 nm. (**B**) TEM image of PM-OMVs-BT. Scale bar = 100 nm. (**C**) NTA results of PM-OMVs. (**D**) NTA results of PM-OMVs-BT.

In this study, PM-OMVs were collected by ultracentrifugation. As shown in Figure 1A, TEM images showed that these particles had a typical bilayer structure. In addition, NTA data showed that the particle size distribution peaks were at 84 nm, 104 nm, and 142 nm (Figure 1B). Unlike mammalian extracellular vesicles (MEVs), OMVs do not have specific protein markers on their surface, such as Alix, CD81, and CD63. The ambiguity of specific markers remains a major challenge for OMVs. Overall, these data indicate that PM-OMVs were successfully isolated.

To construct PM-OMVs-BT, 1 mol Cys-SDSSD and 1 mol DSPE-PEG2000-MAL were mixed and reacted for 24 h to obtain the bone-targeting peptide DSPE-PEG-Mal-Cys-SDSSD. Subsequently, the modified peptide DSPE-PEG 2000-MAL-Cys-SDSSD was coupled to PM-OMVs via hydrophobic insertion. The morphology of PM-OMVs-BT was characterized by TEM and NTA. As shown in (Figure 1C), TEM observation revealed that the particles had a typical double-layer structure. NTA data showed that the particle size distribution peaked at 110 nm (Figure 1D), which was consistent with the particle size of PM-OMVs. These results indicate that the physicochemical engineering approach did not change the size and morphology of PM-OMVs-BT compared with PM-OMVs.

### 3.2. Distribution of PM-OMVs-BT In Vivo

**Figure 2:**
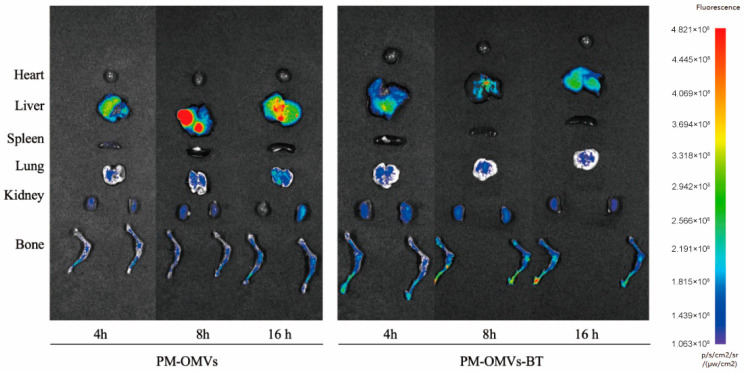
Distribution of PM-OMVs-BT in vivo. Distribution of Cy5-labeled PM-OMVs and PM-OMVs-BT in vivo at 4 h, 8 h, and 16 h after injection.

Osteoporotic bone repair has always been a difficult problem in clinical practice. Although many natural OMVs have been successfully used to treat OP, their use and further promotion are still hindered by the lack of bone-targeting ability. The purpose of bone-targeted therapy is to reduce drug side effects, enhance local drug concentration, and improve treatment efficiency. Engineered OMVs have strong bone-targeting ability and are expected to become a method for the clinical treatment of orthopedic diseases. It has been reported that the SDSSD peptide can bind to periostin and target osteoblasts in the bone microenvironment [26]. Therefore, we evaluated the targeting ability of PM-OMVs-BT in vivo. After intravenous injection of PKH-26-labeled PM-OMVs or PM-OMVs-BT, the bone accumulation of OMVs was evaluated by bio-photonic imaging. The fluorescence of the main organs and femurs of rats in each group was detected at 4 h, 8 h, and 16 h after tail vein injection. As shown in (Figure 2), SDSSD modification can promote the bone accumulation of PM-OMVs-BT and reduce their distribution in other organs. This is consistent with previous studies using SDSSD-modified exosomes and SDSSD-modified polyurethane nano micelles [27]. In conclusion, PM-OMVs-BT are a highly efficient bone-targeted delivery vehicle for bone homeostasis regulation.

### 3.3. Cellular Uptake and Toxicity of PM-OMVs-BT In Vitro

**Figure 3:**
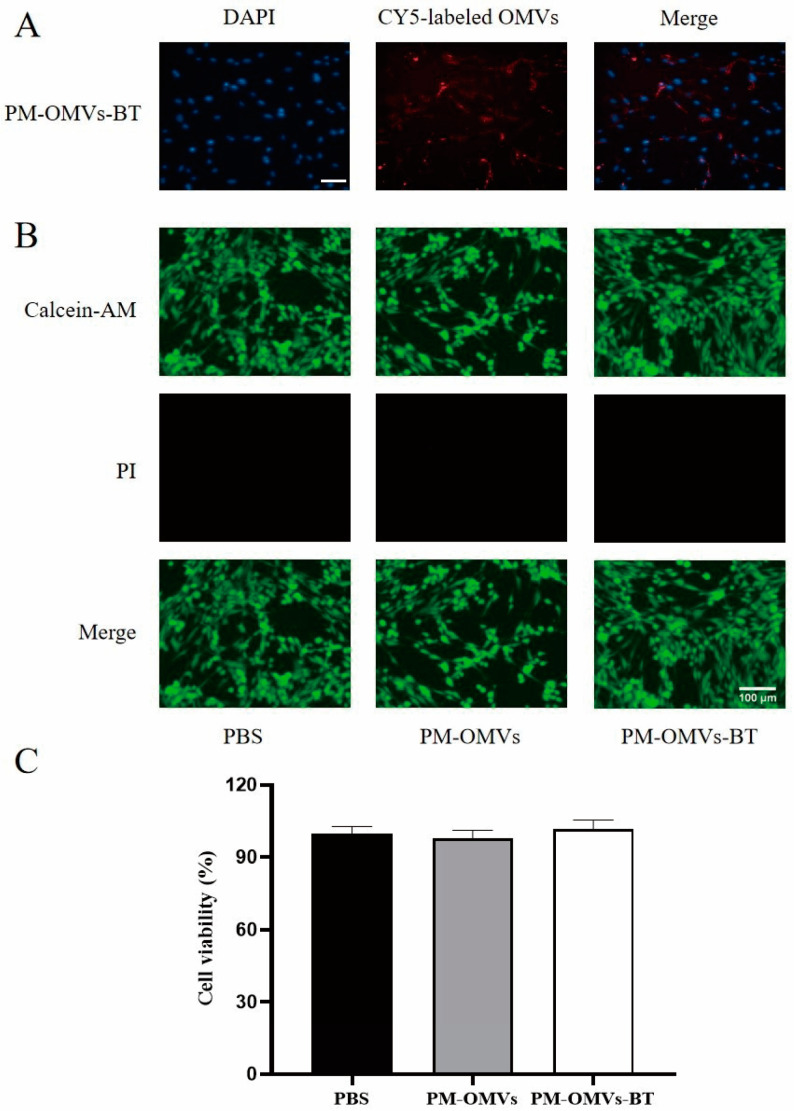
The uptake and cytotoxicity of PM-OMVs and PM-OMVs-BT in vitro: (**A**) Uptake of Cy5-labeled PM-OMVs and PM-OMVs-BT detected by confocal laser scanning microscopy. (**B**) D/L staining after treatment with PBS, PM-OMVs, and PM-OMVs-BT. Scale bar = 100 μm. (**C**) The cytotoxicity of PM-OMVs and PM-OMVs-BT in vitro.

OMVs can be loaded with specific drug molecules, and when they are internalized by the target cell, the drug can be released inside the cell to exert a therapeutic effect. Therefore, we added CY5-labeled PM-OMVs-BT to the cells to probe the internalization process of OMV. By cellular uptake assay, we found that cells significantly internalized a large amount of PM-OMVs-BT (Figure 3A). This result suggests that PM-OMVs-BT can efficiently deliver the contents it carries to target cells. Afterward, we used D/L staining (Figure 3B) and CCK-8 (Figure 3C) to assess the toxicity of PM-OMVs and PM-OMVs-BT on cells. The results of D/L staining showed that PM-OMVs and PM-OMVs-BT-treated cells maintained good viability with no significant effect on cell viability. CCK8 assay further confirmed that the relative proliferation rate of PM-OMVs and PM-OMVs-BT-treated cells remained above 90%, with no significant difference compared to the PBS group. These results indicate that treatment with PM-OMVs and PM-OMVs-BT had no significant cytotoxic effects, and that PM-OMVs-BT are safe at the cellular level.

### 3.4. Toxicity of PM-OMVs-BT In Vivo

**Figure 4:**
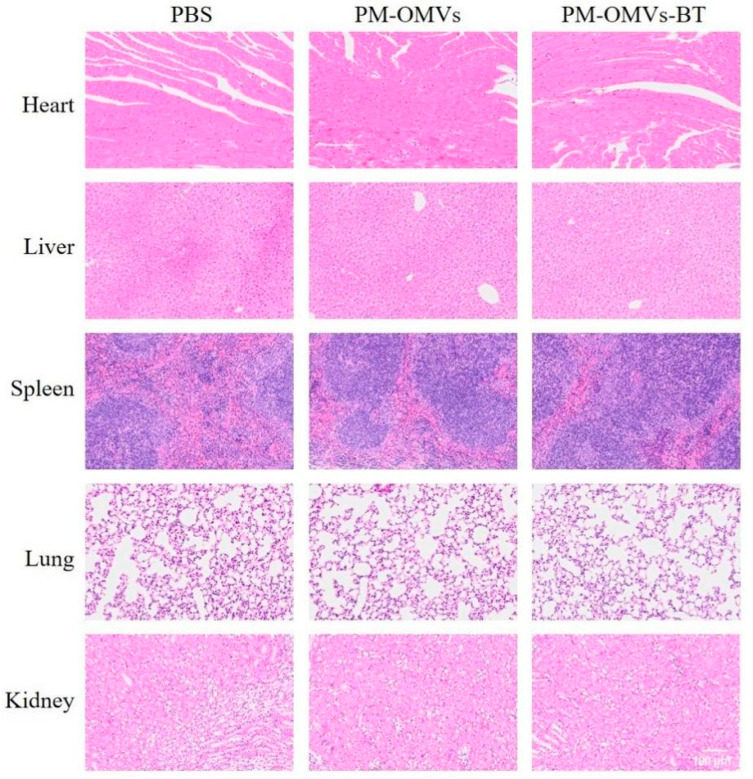
H&E staining of the major organs such as heart, liver, spleen, lung, and kidney after administration of PBS, PM-OMVs, and PM-OMVs-BT. Scale bar = 100 μm.

The in vivo safety of PM-OMVs-BT determines its further applications. We injected PBS, PM-OMVs, and PM-OMVs-BT into healthy mice to check in vivo cytocompatibility. The major organs (heart, liver, spleen, lungs, and kidneys) did not show significant pathological changes in terms of histological structure and cellular morphology compared to the PBS group (Figure 4). This result suggests that PM-OMVs and PM-OMVs-BT exhibit good biocompatibility in mice without acute and systemic toxic reactions. We successfully constructed an engineered PM-OMVs-BT with no obvious toxicity and good biosafety, which lays a solid foundation for subsequent applications.

### 3.5. PM-OMVs-BT Promote Osteogenesis In Vitro

**Figure 5:**
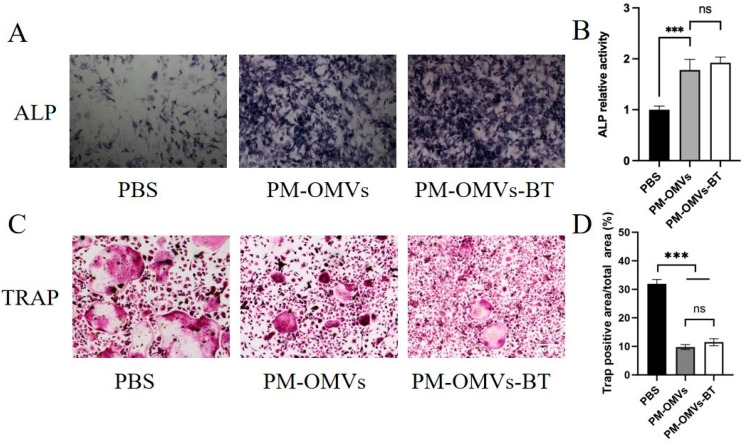
PM-OMVs-BT promote osteogenesis in vitro: (**A**) ALP staining of cells after treatment with PBS, PM-OMVs, and PM-OMVs-BT. Scale bar = 200 μm. (**B**) Quantitative analysis of ALP staining. (**C**) TRAP staining of cells after treatment with PBS, PM-OMVs, and PM-OMVs-BT. Scale bar = 200 μm. (**D**) Quantitative analysis of TRAP staining. *** *p* < 0.001. ns = not significant.

Subsequently, we explored the role of PM-OMVs and PM-OMVs-BT on osteoblasts and osteoclasts during bone repair. The results showed that both PM-OMVs and PM-OMVs-BT could effectively promote osteoblast development, and the ALP level was significantly higher than that of the control group (Figure 5A,B). In addition, by using the TRAP staining method, we found that both PM-OMVs and PM-OMVs-BT significantly reduced osteoclast production (Figure 5C,D). No significant differences were demonstrated between PM-OMVs and PM-OMVs-BT in promoting osteoblasts and inhibiting osteoclast formation. These findings point to the fact that PM-OMVs and PM-OMVs-BT promote the process of bone repair by enhancing osteoblast activity and inhibiting osteoclast formation.

### 3.6. PM-OMVs-BT Alleviate OP In Vivo

**Figure 6:**
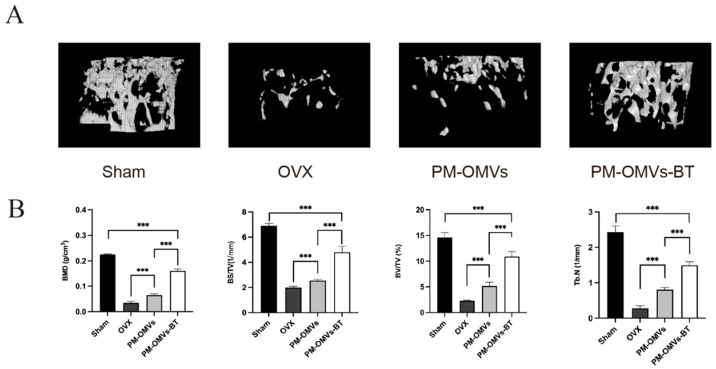
PM-OMVs-BT alleviate OP in vivo: (**A**) Representative micro-CT images of the distal femur r in Sham group, OVX group, OVX mice treated with PM-OMVs (PM-OMVs group), and OVX mice treated with PM-OMVs-BT (PM-OMVs-BT group). (**B**) Quantitative analysis of BMD, BV/TV, BS/TV, and Tb. N, respectively. *** *p* < 0.001.

To investigate the effect of PM-OMVs-BT on OP in vivo, an ovariectomized (OVX) mouse model was established. OVX mice were randomly divided into three groups and intravenously injected with PBS, PM-OMVs, and PM-OMVs-BT, respectively. OVX mice were administered once a week for 8 weeks, and then euthanized to collect distal femurs. As shown in Figure 6A, after treatment with PM-OMVs and PM-OMVs-BT, bone loss gradually slowed down, and the number and volume of trabeculae gradually increased. Further quantitative analysis of bone microstructure confirmed the therapeutic effect of PM-OMVs-BT on OP. As shown in the Figure 6B, the bone microarchitecture of the PM-OMVs and PM-OMVs-BT groups was enhanced, including bone mineral density (BMD), bone volume fraction (BV/TV), bone surface area/total volume (BS/TV), and trabecular number (Tb.N), which were all higher than those of the OVX group. It is worth noting that the bone microarchitecture of the PM-OMVs-BT group was better than that of the non-targeted PM-OMVs group, indicating that bone targeting is of great significance in the treatment of OP.

## 4. Discussion

Current treatments for OP often face limitations such as poor patient compliance, side effects, and incomplete restoration of bone quality. Therefore, the development of new therapeutic strategies is of great significance. In this study, we explored a novel approach by developing a bone-targeted delivery system based on engineered PM-OMVs-BT to improve the treatment outcomes of OP.

Our findings indicated that bone-targeted OMVs can significantly enhance the osteogenic differentiation of BMSCs while suppressing osteoclast formation. These dual actions are crucial for combating OP, as they address both the anabolic and catabolic aspects of bone metabolism. Specifically, enhancing osteogenesis through BMSCs differentiation helps in the formation of new bone tissue, while inhibiting osteoclastogenesis prevents excessive bone resorption. Consequently, this bone-targeted delivery system exhibited a promising therapeutic effect on OP in both in vitro and in vivo experiments, suggesting its potential as a novel treatment strategy.

This discovery not only provides a new avenue for the treatment of OP but also significantly enriches the research on the gut–bone axis based on microbial OMVs. The gut–bone axis is an emerging field that explores the interactions between the gut microbiota and bone health. OMVs, which are nanoscale vesicles released by bacteria, have garnered increasing attention due to their ability to deliver bioactive molecules and modulate host cellular responses. By engineering PM-OMVs-BT to target bone tissue, we have expanded the application scope of microbial OMVs in skeletal health and disease.

In our in vitro experiments, both non-targeted OMVs and targeted OMVs were added to the culture medium. However, due to the lack of a physiological environment, the significance of targeting was not fully reflected in these settings. The absence of a bone-specific targeting mechanism meant that the therapeutic effects of non-targeted OMVs and targeted OMVs were similar in vitro. Cui et al. [27] also achieved good targeting in vivo using SDSSD peptide. This highlights the importance of evaluating the targeting efficiency in a more biologically relevant context.

Moreover, the consistency of OMVs across different batches is a critical factor that may affect their clinical translation. Variations in the composition, size, or functional properties of OMVs between batches could lead to inconsistent therapeutic outcomes. In large-scale production and clinical applications, it is imperative to ensure that each batch of OMVs maintains the same therapeutic effect and safety profile. Achieving batch-to-batch consistency requires further optimization of the production process, including the selection of appropriate bacterial strains, culture conditions, and purification methods. Additionally, the establishment of a robust quality control system is essential to monitor and ensure the consistency and quality of OMVs. This system should include standardized protocols for assessing the physicochemical properties, biological activity, and safety of OMVs in each batch.

Furthermore, while our short-term experiments have shown the good safety and therapeutic effects of OMVs, their long-term efficacy and potential side effects remain to be fully evaluated. Long-term exposure to OMVs may lead to unforeseen consequences, such as immune responses, tissue toxicity, or alterations in the microbiota–host interactions. Therefore, it is necessary to conduct more extensive long-term animal experiments to assess the durability of the therapeutic effects and to monitor for any delayed adverse effects. Additionally, well-designed clinical trials should be carried out to evaluate the safety and efficacy of bone-targeted OMVs in human subjects. These trials should include a diverse patient population and should monitor for both immediate and long-term outcomes to provide a comprehensive assessment of the therapeutic potential of this delivery system.

## 5. Conclusions

This study developed a delivery system using PM-OMVs, enhanced with bone-targeting peptides to deliver miRNAs to bone effectively. These bone-targeted PM-OMVs showed exceptional targeting ability and safety in vivo. Additionally, they promoted osteogenic differentiation of BMSCs without significant cytotoxicity. The findings suggest that PM-OMVs-BT could be a safe and effective new treatment for OP and enrich the research on the gut–bone axis based on OMVs.

## Data Availability

Datasets are available on request: The raw data supporting the conclusions of this article will be made available by the first authors and corresponding author, without undue reservation.

## Abbreviations

Osteoporosis (OP); extracellular vesicles (EVs); outer membrane vesicles (OMVs); *pseudomonas mirabilis* (PM); bone-targeted PM-OMVs (PM-OMVs-BT); Bone targeting LGG-EVs (BT-LGG-EVs); 1,2-Distearoyl-sn-glycero-3-phosphoethanolamine (DSPE); Maleimide (Mal); bone marrow stromal cells (BMSCs); short-chain fatty acids (SCFAs); *Lactobacillus rhamnosus* GG (LGG); transmission electron microscopy (TEM); nanoparticle tacking analysis (NTA); fetal bovine serum (FBS); alkaline phosphatase (ALP); three-dimensional (3D); mammalian extracellular vesicles (MEVs); ovariectomized (OVX); bone mineral density (BMD); bone volume fraction (BV/TV); bone surface area/total volume (BS/TV); trabecular number (Tb.N)
